# N6-methyladenosine RNA modification (m6A) is of prognostic value in HPV-dependent vulvar squamous cell carcinoma

**DOI:** 10.1186/s12885-022-10010-x

**Published:** 2022-09-01

**Authors:** Mateja Condic, Thore Thiesler, Christian Staerk, Niklas Klümper, Jörg Ellinger, Eva K. Egger, Kirsten Kübler, Glen Kristiansen, Alexander Mustea, Damian J. Ralser

**Affiliations:** 1 Department of Gynecology and Gynecological Oncology, University Hospital Bonn, Bonn, Germany; 2 Institute of Pathology, University Hospital Bonn, Bonn, Germany; 3 Department of Medical Biometry, Informatics and Epidemiology, University Hospital Bonn, Bonn, Germany; 4 Department of Urology and Pediatric Urology, University Hospital Bonn, Bonn, Germany; 5 Berlin Institute of Health at Charité – Universitätsmedizin Berlin, Charitéplatz 1, 10117 Berlin, Germany; 6 Department of Hematology, Oncology and Cancer Immunology, Charité – Universitätsmedizin Berlin, corporate member of Freie Universität Berlin and Humboldt-Universität Zu Berlin, Hindenburgdamm 30, 12203 Berlin, Germany

**Keywords:** m6A, N6-methyladenosine RNA modification, Vulvar squamous cell carcinoma, HPV, Biomarker

## Abstract

**Background:**

Vulvar squamous cell carcinoma (VSCC) is an uncommon gynecologic malignancy but with an increasing incidence in recent years. Etiologically, VSCC is classified into two subtypes: HPV-dependent and HPV-independent. Localized VSCC is treated surgically and/or with radiation therapy, but for advanced, metastatic or recurrent disease, therapeutic options are still limited.

N6-methyladenosine (m6A) is the most prevalent post-transcriptional messenger RNA (mRNA) modification and involved in many physiological processes. The group of m6A proteins can be further divided into: ‚writers’ (METTL3, METTL4, METTL14, WTAP, KIAA1429), ‚erasers’ (FTO, ALKBH5), and ‚readers’ (HNRNPA2B1, HNRNPC, YTHDC1, YTHDF1-3). Dysregulated m6A modification is implicated in carcinogenesis, progression, metastatic spread, and drug resistance across various cancer entities. Up to date, however, only little is known regarding the role of m6A in VSCC.

**Methods:**

Here, we comprehensively investigated protein expression levels of a diverse set of m6A writers, readers and erasers by applying immunohistochemical staining in 126 patients with primary VSCC.

**Results:**

In the entire study cohort, dominated by HPV-independent tumors, m6A protein expression was not associated with clinical outcome. However, we identified enhanced protein expression levels of the ‚writers’ METTL3, METTL14 and the ‚reader’ YTHDC1 as poor prognostic markers in the 23 patients with HPV-dependent VSCC.

**Conclusion:**

Our study suggests dysregulated m6A modification in HPV-associated VSCC.

**Supplementary Information:**

The online version contains supplementary material available at 10.1186/s12885-022-10010-x.

## Background

Vulvar carcinoma is responsible for 3% of all gynecological malignancies worldwide and represents the fourth most common tumor of the female genital tract [1]. In the last decade, the incidence of human papillomavirus (HPV)-dependent and HPV-independent vulvar carcinoma has increased by more than 20%, likely driven by increased high-risk HPV exposure and a generally aging population [2–4].

Vulvar squamous cell carcinoma (VSCC) is the predominant histological subtype. VSCC can be further sub-classified into two etiologic subtypes: (i) HPV-dependent VSCC [5, 6], accounting for 34% of invasive VSCC [7]; and (ii) HPV-independent VSCC arising on the basis of *lichen sclerosus and atrophicus* [8], a chronic vulvar dermatosis affecting mostly elderly patients. Of note, HPV-independent VSCC displays a worse overall prognosis than HPV-dependent VSCC [9].

In localized disease, tumor excision with inguinofemoral sentinel lymphonodectomy and/or inguinofemoral systematic lymphonodectomy represents the therapeutic mainstay. Additional radiotherapy is applied in the presence of risk factors. With > 85% survival rates, the 5-year overall survival (OS) is excellent in localized disease [10]. However, for patients with locally advanced, metastatic or recurrent disease, there are only limited treatment strategies with an overall poor 5-year OS of only 15–50% [11]. Hence, there is an unmet need for new therapeutic options in this difficult-to-treat patient population [12]. In this context, a deeper understanding of the VSCC tumor biology, in particular for the respective etiologic subtypes, might pave the way to identify novel therapeutic approaches in VSCC.

N6-methyladenosine (m6A) is the most abundant messenger RNA (mRNA) modification. Briefly, three different enzyme groups are involved in m6A modification: (i) methylases (‘writers’; METTL 3, METTL 4, METTL 14, WTAP, KIAA1429) that catalyze the transfer of S-adenosyl methionine groups to RNA adenine bases; (ii) demethylases (‘erasers’; FTO, ALKBH5) that have the capacity to reverse the methylation process; and (iii) ‘readers’ (HNRNPA2B1, HNRNPC, YTHDC1, YTHDF1-3) that recognize m6A RNA modification and activate downstream regulatory pathways [13].

m6A modifications were previously identified to be involved in tumorigenesis, proliferation, angiogenesis and tumor immunity across diverse cancer entities [14–20]. This central role of m6A emphasizes its great potential in both diagnostic and therapeutic applicability [21]. Recently, we were able to provide evidence for m6A involvement in CC that bears etiological and tumor biological similarity to VSCC [22]. To the best of our knowledge, there is no data regarding m6A modification in VSCC. In this study, we thus comprehensively analyzed protein expression levels of a diverse set of m6A writers, readers and erasers by immunohistochemistry in a cohort of 126 VSCC patients to understand the effects of RNA modifications on tumorigenesis, especially with regard to the two etiologic subtypes.

## Methods

### Patients and specimens

The retrospective single-center study population included 126 patients with primary VSCC treated at the University Hospital Bonn between 2002 and 2017. The collection of tissue was within the framework of the Biobank initiative of the University Hospital Bonn. Tissue was obtained from biopsies or surgical specimens. All patients provided written informed consent prior to the collection of biomaterials. The study was approved by the Ethics Committee of the Medical Faculty of the University of Bonn (vote: 208/21).

Clinicopathological characteristics of the entire cohort, the HPV-independent and the HPV-dependent sub-cohorts, obtained from a clinical database, are presented in Table 1. The histopathological diagnosis was based on the World Health Organization (WHO) criteria. The 2010 revision of the International Federation of Gynecology and Obstetrics (FIGO) system was used to determine the tumor grade. The 7th TNM classification of the Union for International Cancer Control (UICC) allowed to determine the tumor stage.

**Table 1:** Clinicopathological characteristics of the entire VSCC cohort, HPV-independent cohort, and HPV-dependent sub-cohorts. No HPV status was available for 24 patients. SD = standard deviation

**Clinicopathological parameters**	**All** (*N* = 126)	**HPV-independent** (*N* = 79)	**HPV-dependent** (*N* = 23)
**Age (years)**			
*Mean (*± *SD)*	64.1 ± 14.4	65.1 ± 14.0	57.5 ± 15.2
*Min–max*	25—93	33—93	25—84
**Overall survival (months)**			
Mean *(*± *SD)*	54.0 ± 42	58.8 ± 44.7	53.7 ± 39.5
Median	46.0	58.0	48.0
**TNM classification**			
T1	102 (81.0%)	65 (82.3%)	16 (69.6%)
T2	17 (13.5%)	12 (15.2%)	4 (17.4%)
T3	3 (2.4%)	1 (1.3%)	2 (8.7%)
Tx	4 (3.2%)	1 (1.3%)	1 (4.3%)
N0	48 (38.1%)	33 (41.8%)	4 (17.4%)
N1	10 (7.9%)	6 (7.6%)	4 (17.4%)
N2	21 (16.7%)	16 (20.3%)	4 (17.4%)
N3	1 (0.8%)	0	0
Nx	46 (36.5%)	24 (30.4%)	11 (47.8%)
**Grading**			
G1	11 (8.7%)	5 (6.3%)	2 (8.7%)
G2	82 (65.1%)	52 (65.8%)	16 (69.6%)
G3	29 (23.0%)	19 (24.1%)	4 (17.4%)
not determined	4 (3.2%)	3 (3.8%)	1 (4.3%)
**HPV-subtypes**			
16			18 (76%)
33			3 (12%)
33 + 16			3 (12%)

### Tissue microarray (TMA) construction

The TMA was generated from formalin-fixed paraffin (FFPE)-embedded VSCC tissue specimens. Hematoxylin and eosin (HE) stained sections were applied to identify representative tumor areas. Subsequently, a 1 mm core biopsy (0.785mm^2^) was taken from the selected cancer areal and arranged in TMA blocks.

### DNA extraction und HPV analysis

Tumor tissue was deparaffinized and macrodissected from unstained slides. The tumor tissue was then lysed with proteinase K overnight. DNA extraction from FFPE-embedded tissue was performed with the BioRobot M48 Robotic workstation and the corresponding MagAttract DNA Mini M48 Kit (Qiagen, Germany). Determination of HPV subtypes was performed applying the HPV Type 3.5 LCD-Array Kit (Chipron, Germany) according to the manufacturer’s instructions as described previously [23]. With this assay the detection of 32 different HPV subtypes is possible (HPV types 06,11,16,18,31,33,35,39, 42,44,45,51,52,53,54,56, 58,59,61,62,66,67,68,70, 72,73,81,82,83,84,90 and 91).

### Immunohistochemistry

Immunostaining of METTL3, METTL4, METTL14, WTAP, KIAA1429, FTO, ALKBH5, HNRNPA2B1, HNRNPC, YTHDC1, YTHDF1,YTHDF2, and YTHDF3 was performed on the TMAs using an automated staining system (BenchMark ULTRA; Ventana Medical Systems) which performed deparaffinization, pretreatment with cell conditioning buffer (CC1 buffer, pH8), and incubation with primary antibodies (FTO (1:50; Atlas Antibodies #HPA041086), ALKBH5 (1:200; Novus #NBP1-82,188), METTL3 (1:1000; Biorbyt #orb374082), METTL4 (1:40; Atlas Antibodies #HPA040061), METTL14 (1:100; Atlas Antibodies #HPA038002), WTAP (1:100; Atlas Antibodies #HPA010550), KIAA1429 (1:25; Atlas Antibodies #HPA031530), HNRNPC (1:25; Atlas Antibodies #HPA051075), HNRNPA2B1 (1:100; Atlas Antibodies #HPA001666), YTHDC1 (1:25; Atlas Antibodies #HPA036462), YTHDF1 (1:10; Biorbyt #orb179018), YTHDF2 (1:200; Biorbyt #orb39199), YTHDF3 (1:200; Biorbyt #orb374095) at 4 °C overnight. Signal detection was performed with the UltraView DAB IHC Detection Kit (Ventana).

Immunostained cells were analyzed with an Olympus BX51 microscope and the Panoramic Viewer 3DHistech. Staining intensities were evaluated for all m6A proteins separately by MC and DJR. In case of discordance between these two investigators, TT was consulted as a board-certified gynecopathologist. In addition, random reviews of the staining intensities were conducted by TT. In detail, a four-tier scoring system was applied to categorize staining intensities (0: no staining, 1: low staining, 2: moderate staining, 3: high staining). Staining intensities were divided into two groups (low and high expression) based on the median protein expression in the entire study cohort.

### Statistical analysis

Kaplan–Meier survival analyses and log-rank tests allowed to compare OS between the two groups (low vs. high expression) for each analyzed protein. Correlation analyses were performed applying the nonparametric Mann–Whitney U test. In addition, the two-sided Fisher's exact test was used for the evaluation of statistical significance; a significance threshold was considered at a *p*-value of < 0.05. We performed multiple hypotheses testing using the method of Benjamini and Hochberg and converted *p*-values to false discovery rate (FDR) *q*-values with a significance threshold of *q* < 0.1. Non-parametric Spearman’s *p* correlation coefficients were calculated for co-expression analysis. Statistical analysis was performed with the Statistical Package for the Social Sciences (SPSS ®) version 28 (SPSS Inc., IBM Corp.) and the GraphPad Prism software (GraphPad software).

## Results

### Proteins of m6A are frequently expressed in VSCC

Across the cohort of 126 primary VSCC samples (Table 1) we identified protein expression of all distinct m6A writers, readers and erasers. The proteins involved in the different m6A functions were present in the different cell compartments reflecting the diversity of RNA metabolism. Writers were typically observed in the nucleus including METTL3, METTL14, WTAP and KIAA1429. Likewise, immunohistochemical analysis revealed a strong nuclear staining for the eraser FTO, and the two readers HNRNPC und HNRNPA2B1. In contrast, the readers YTHDF1, YTHDF2 and YTHDF3 as well as the writer METTL4 showed a strong cytoplasmic staining (Table 2).

**Table 2:** Summary of the analyzed m6A proteins as indicated and their correlation with overall survival (indicated as %alive) for the entire study cohort, HPV-independent, and HPV-dependent VSCC. The HPV-status was not available for 24 patients. Samples were grouped according to high and low expression based on the staining intensities. *p*-values for the group comparisons are based on log-rank tests (significance threshold *p* < 0.5). *q*-values are based on multiple hypotheses testing using the method of Benjamini and Hochberg with a significance threshold of q < 0.1

			**All**			**HPV-independent**			**HPV-dependent**			
**Proteins**	**Localisation**	**Staining intensity** (low/high)	N (low/high)	%alive (low/high)	*p*-value	N (low/high)	%alive (low/high)	*p*-value	N (low/high)	%alive (low/high)	*p*-value	*q-* value
**Writer**												
METTL3	nuclear	0–2 + /3 +	77/27	62.3/40.7	0.104	51/18	56.9/38.9	0.435	11/7	81.8/28.6	**0.010**	**0.08**
METTL4	cytoplasmatic	0–1 + /2–3 +	60/36	58.3/50.0	0.113	44/24	54.5/45.8	0.128	8/7	87.5/42.9	**0.034**	0.10
METTL14	nuclear	0–1 + /2–3 +	54/45	55.6/55.6	0.586	44/23	47.7/60.9	0.541	6/10	100/55.6	**0.020**	**0.09**
WTAP	nuclear	0–2 + /3 +	90/16	54.4/68.8	0.311	63/6	49.2/83.3	0.132	16/4	68.8/75.0	0.694	0.74
KIAA1429	nuclear	0–1 + /2–3 +	66/35	56.1/60.0	0.782	47/22	53.2/59.1	0.661	10/5	60.0/40.0	0.482	0.70
**Eraser**												
FTO	nuclear	0–2 + /3 +	83/21	55.4/76.2	0.113	61/9	54.1/66.7	0.361	10/9	50.0/77.8	0.257	0.48
ALKBH5	cytoplasmatic/ nuclear	0–1 + /2–3 +	44/63	50.0/65.1	0.231	35/35	45.7/62.9	0.161	4/15	50.0/66.7	0.699	0.74
**Reader**												
HNRNPA2B1	nuclear	0–2 + /3 +	85/23	57.6/73.9	0.429	62/10	54.8/70.0	0.602	17/2	58.8/100	0.297	0.48
HNRNPC	nuclear	0–1 + /2–3 +	54/48	51.9/62.5	0.162	34/35	50.0/54.3	0.518	11/7	63.6/71.4	0.736	0.74
YTHDC1	membraneous/ cytoplasmatic/ nuclear	0–2 + /3 +	78/23	61.5/43.5	0.111	58/12	58.6/41.7	0.496	12/7	83.3/28.6	**0.012**	**0.08**
YTHDF1	cytoplasmatic	0–2 + /3 +	70/26	57.1/50.0	0.893	52/15	55.8/33.3	0,409	10/4	60.0/75.0	0.551	0.72
YTHDF2	cytoplasmatic	0–1 + /2–3 +	31/73	58.1/54.8	0.422	21/48	52.4/52.1	0.665	5/13	100/46.2	**0.040**	0.10
YTHDF3	cytoplasmatic	0–1 + /2–3 +	45/51	55.6/58.8	0.725	28/37	50.0/56.8	0.554	6/7	66.7/57.1	0.084	0.18

### Proteins of m6A are differently expressed in VSCC subtypes

Given the two etiologically distinct VSCC subgroups, namely HPV-dependent and HPV-independent VSCC, each subgroup was next examined separately. In the HPV-dependent subgroup of 23 patients, 76% of cases were positive for HPV type 16, 12% for HPV type 33, and 12% displayed a co-infection with both HPV types 16 and 33. The HPV-independent cohort comprised 79 patients. For 24 patients, HPV status was unknown (Table 1).

**Fig. 1:**
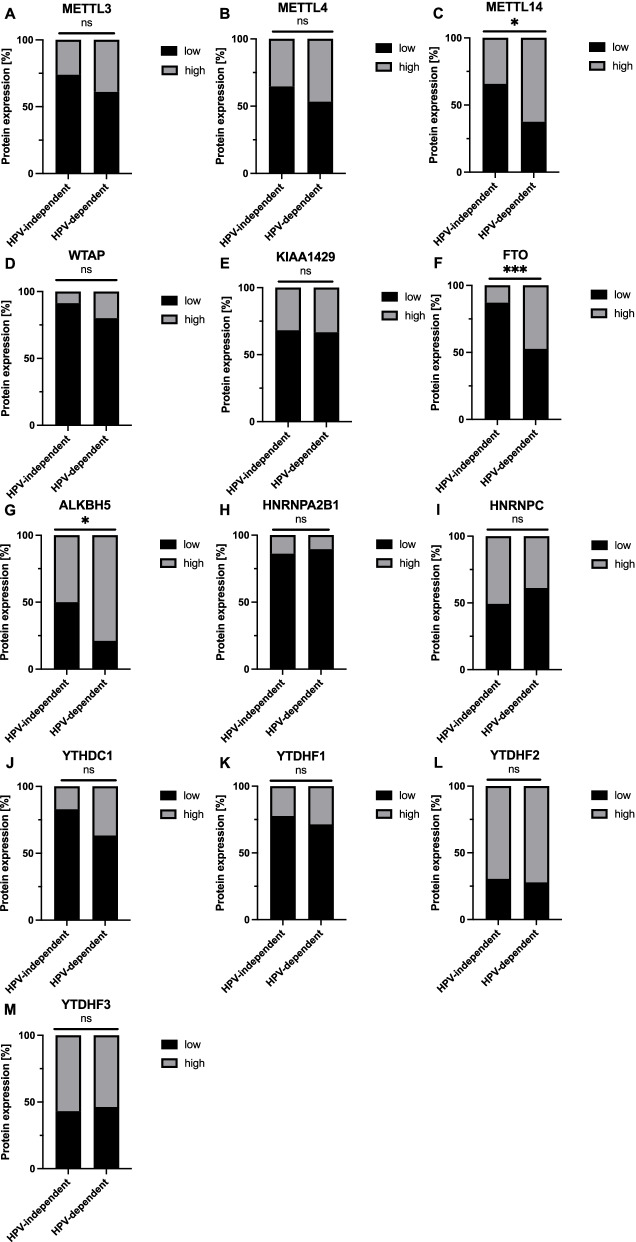
Differential expression (high vs. low) of m6A protein depending on the VSCC subtype. * *P* < 0.05; ** *P* < 0.01; *** *P* < 0.001 (Fisher’s exact test)

First, we analyzed m6A proteins for their different expression regarding to the VSCC subtypes. For most m6A proteins (10/13), we did not find a differential expression between the two etiologic subtypes (Fig. 1A-B, D-E, H-M). However, we observed differences for 3 proteins that were all significantly enriched in HPV-dependent VSCC: the writer METTL14 (63% vs 34% in HPV-independent VSCC; *p* = 0.049, Fisher's exact test; Fig. 1C), and the erasers FTO (47% vs 13% in HPV-independent VSCC; *p* = 0.002, Fisher's exact test; Fig. 1F), and ALKBH5 (79% vs 59% in HPV-independent VSCC; *p* = 0. 040, Fisher's exact test; Fig. 1G).

### Proteins of m6A indicate poor outcome in HPV-dependent but not HPV-independent VSCC

**Fig. 2:**
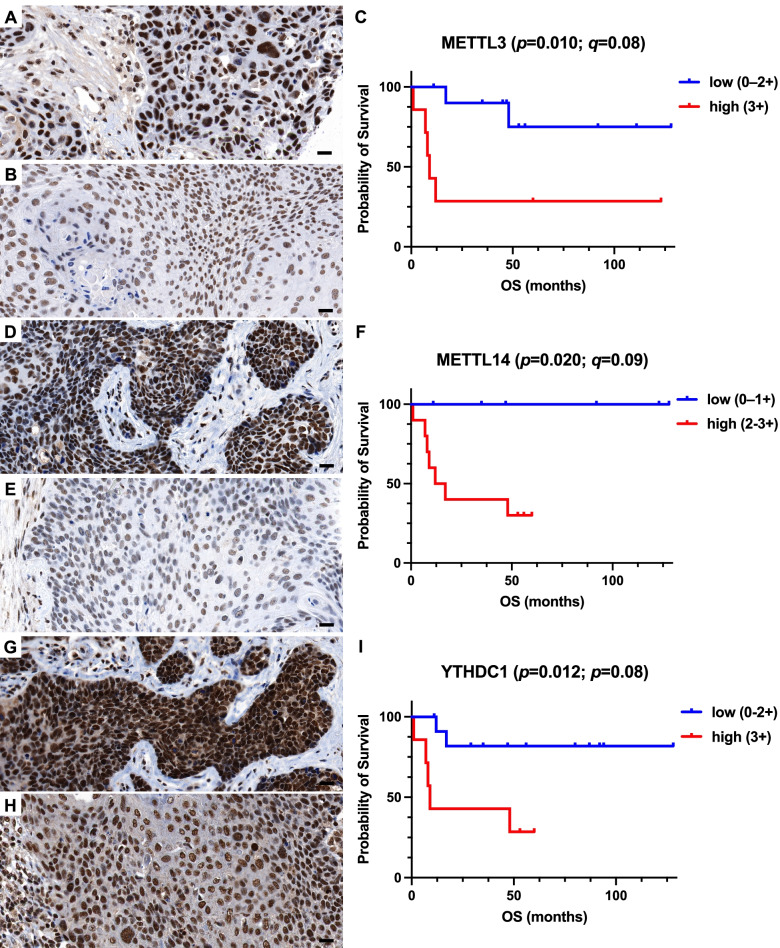
Representative histology sections show high (**A**, **D**, **G**) and low (**B**, **E**, **H**) expression levels of METTL3, METTL14 and YTHDC1 visualized by immunohistochemistry; hematoxylin (blue) was used for nuclear staining (bright field image, 400xmagnification). Kaplan–Meier estimates show a significantly shorter 5-year survival (*p* < 0.05) in patients with high expression of METTL3, (**F**) METTL14, and (I) YTHDC1. Prognostic significance remained after correction for multiple testing (*q* < 0.1). Scale bar = 20 um

In the entire cohort, none of m6A proteins analyzed was associated with OS **(**Table 2**)**. Likewise, when focusing our analysis on HPV-independent VSCCs only, we also did not find an association with outcome (Table 2). However, when evaluating the subgroup of HPV-dependent VSCC, high expression levels of the writers METTL3 (*p* = 0.010, *q* = 0.08, log-rank test and Benjamini and Hochberg corrected log-rank test; Fig. 2A-C; Table 2), METTL 14 (*p* = 0.020, *q* = 0.09, Fig. 2D-F) and the reader YTHDC1 (*p* = 0.012, *q* = 0.08, F ig. 2G-I) were significantly correlated with shorter OS. Increased expression of the writer METTL4 (*p* = 0.034, Supplementary Fig. 1A-C) and the reader YTHDF2 (*p* = 0.040, Supplementary Fig. 1D-F) were also associated with poor outcome but did not remain significant when correcting for multiple hypothesis testing at a significance threshold of *q* < 0.1. Protein expression levels of METTL3, METTL4, METTL14, YTDHC1, and YTHDF2 were not associated with the clinicopathological parameters nodal stage and histomorphological grading in the entire study cohort and the two subgroups, respectively (Supplementary Table 1).

**Fig. 3:**
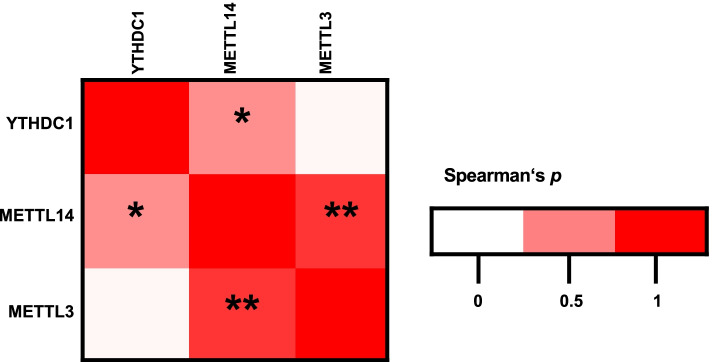
Correlation heatmap visualizes Spearman’s *p* correlation coefficients of METTL3, METTL14, and YTDHC1 in the HPV-dependent VSCC cohort. * *P* < 0.05; ** *P* < 0.01 (two-sided t-test)

We found high positive Spearman’s *p* correlation coefficients for the expression of the prognostic m6A proteins METTL3, METTL14, and YTDHC1, respectively in the HPV-dependent subgroup (Fig. 3). In 6/15 patients, high levels of the writers METTL3 and METTL14 were cooccurring (Spearman’s *p* = 0.797; two-sided t-test *p* =  < 0.001). Likewise, in 5/15 patients the writer METTL3 and the reader YTHDC1 (Spearman’s *p* = 0.036; two-sided t-test *p* = 0.872) and in 7/15 patients the writer METTL14 and YTHDC1 (Spearman’s p = 0.443; two-sided t-test *p* = 0.034) were at high levels. Of these 3 proteins, we identified METTL14 to be the protein that typically cooccurred with the others in contrast to METTL3 and YTHDC1 that gave additional information to the other two.

## Discussion

In the present study, our analysis suggests that expression levels of the ‘writers’ METTL3 and METTL14 and the ‘reader’ YTHDC1 are involved in HPV-dependent VSCC tumorigenesis, but not HPV-independent tumor development. HPV is a small DNA virus that is usually transmitted sexually. Sexually active individuals carry a lifetime risk for HPV infection of around 80–90% [24]. It is estimated, that 5% of human cancers are caused by a persistent infection with high risk HPV types [25] including not only VSCC but also cervical, penile, and head and neck SCC [26]. HPV-dependent VSCC account for 30% of all VSCC cases and exhibit a more favorable prognosis compared to the HPV-independent VSCC subtype. Although HPV-dependent and HPV-independent VSCC represent etiologically different subtypes, both are treated equally in current clinical practice [27].

Previous research has shown that m6A modification is implicated in viral infection by modulating the interaction between the virus and the host. Thus, m6A can influence both, the susceptibility of the host cells to viral infection, and the replication of the virus in the host cell [28, 29]. There is only sparse known regarding m6A modification in the context of HPV infection and cancer. In cervical cancer (CC), that is predominantly caused by infection with high-risk HPV, there is broad scientific evidence, that abnormal m6A modification plays an essential role in tumor proliferation, angiogenesis and metastatic spread. METTL3 was shown to be upregulated in CC cells and linked to lymph node metastasis and unfavorable outcomes [30]. Further, m6A dysregulation is linked to chemo- and radiotherapy-resistance and a more progressive CC phenotype [17]. In this context, especially the demethylase FTO was identified to be an important oncogenic driver by regulating proliferation and migration of CC cells [31]. Another study confirmed the importance of METTL14 in CC. Silencing METTL14 induced a cell cycle arrest in CC cells via the the PI3K/AKT/mTOR signaling pathway [32]. The interaction between m6A and the PI3K/AKT/mTor signaling pathway has also been described for endometrial cancer and further entities [16, 33]. The etiologic resemblance of CC and VSCC suggests dysregulated m6A modification to be involved in VSCC tumorigenesis. In head and neck SCC, which are frequently associated with HPV-infection, overexpression of METTL3 and METTL14 correlated with advanced T stage and poor OS [34]. Further, enhanced METTL3 expression was observed in oral SCC, that is also linked to HPV infection [35].

There is no data available regarding the precise biological mechanism of m6A modification and HPV-driven tumorigenesis. However, there is data on other oncogenic viruses like Kaposi’s sarcoma-associated herpesvirus (KSHV): Research has shown, that depletion of METTL3 and YTHDF2 lead to lower expression levels of the lytic genes ORF50 and ORF57 as well as decreased virion production [36]. Lytic genes are required to enter the viral lytic replication cycle. These findings suggest m6A to promote a pro-viral environment for KSHV infection. Comparable data were obtained for simian virus 40. Here, overexpression of YTHDF2 was found to be associated with enhanced viral replication in BSC40 cells whereas depletion of YTHDF2 or METTLL3 lead to contrary effects [37].

Besides METTL3 and METTL14, our analysis also showed significant data for the reader YTHDC1 in the HPV-dependent VSCC subgroup. As YTHDF2, YTHDC1, is involved in mRNA splicing, nuclear export and translation. In the context of viral infection, research has shown, that YTHDC1 is involved in splicing of genes important for the lytic replication [36]. Given the involvement of m6A in HPV-dependent VSCC harbors the potential to be used therapeutically. 3-deazaadenosine (DAA) inhibits m6A modification and has exhibit antiviral effects in both, cell culture and mouse models of viral infection [38]. To date, it has not been studied whether there is also cytotoxic potential of DAA in HPV-dependent malignancies. In addition to direct drug targeting of methylation, inhibition of the PIK3/AKT/mTOR signaling pathway might be a promising therapeutic option, in particular due to the described interaction between m6A and this pathway. There are various therapeutic agents that could be considered, such as everolimus or the PIK3 inhibitor alpelisib. So far, these therapeutics have not been investigated in VSCC, but, however, might be of potential interest.

Our findings point towards the important role of m6A RNA modification in cancer and especially in HPV-dependent tumors. This is the first study implicating the relationship between HPV infection, m6A RNA modification, and carcinogenesis in VSCC. However, as a limitation of the present study, the relatively small cohort size of 23 HPV-dependent VSCC has to be mentioned. Consecutively, multivariate statistical analyses could not be performed. A further limitation is the retrospective study design and the determination of protein expression based on a tissue microarray with sigle cores per sample. Hence, tumor heterogeneity might not be adequately reflected by our method approach. However, to the best of our knowledge, there is no evidence for intratumoral heterogeneity regarding m6A protein expression analysis. Of note this is also reflected by our own data regarding m6A protein expression in endometrial and cervical cancer [22, 39].

Dysregulation of m6A proteins might be used as biomarkers and indicators for poor prognosis but also as potential targets for novel therapeutic drugs. However, the specific mechanisms explaining the interaction of m6A modification and HPV infection remains to be elucidated in further studies.

## Conclusion

High expression levels of proteins involved in m6A modification correlate with a poor OS in patients with HPV-dependent VSCC. Hence, m6A might serve as a prognostic biomarker in HPV-dependent VSCC.

## Supplementary Information

**Additional file 1.  **

**Additional file 2.**

## Data Availability

The datasets generated and/or analyzed during the current study are available on request from the authors.

## Abbreviations

CC: Cervical cancer
FDR: False discovery rate
FFPE: Formalin-fixed paraffin-embedded
FIGO: International Federation of Gynecology and Obstetrics
HE: Hematoxylin and eosin
HPV: Human papillomavirus
KSHV: Kaposi’s sarcoma-associated herpesvirus
m6A: N6-methyladenosine (m6A)
mRNA: Messenger RNA
OS: Overall survival
SCC: Squamous cell carcinoma
TMA: Tissue microarray
UICC: Union for International Cancer Control
VSCC: Vulvar squamous cell carcinoma
WHO: World Health Organization (WHO)

## References

[1] Sankaranarayanan R, Ferlay J (2006). Worldwide burden of gynaecological cancer: the size of the problem. Best Pract Res Clin Obstet Gynaecol.

[2] Judson PL, Habermann EB, Baxter NN, Durham SB, Virnig BA (2006). Trends in the incidence of invasive and in situ vulvar carcinoma. Obstet Gynecol.

[3] Kang YJ, Smith M, Barlow E, Coffey K, Hacker N, Canfell K (2017). Vulvar cancer in high-income countries: Increasing burden of disease. Int J Cancer.

[4] Schuurman MS, van den Einden LC, Massuger LF, Kiemeney LA, van der Aa MA, de Hullu JA (2013). Trends in incidence and survival of Dutch women with vulvar squamous cell carcinoma. Eur J Cancer.

[5] Insinga RP, Liaw KL, Johnson LG, Madeleine MM (2008). A systematic review of the prevalence and attribution of human papillomavirus types among cervical, vaginal, and vulvar precancers and cancers in the United States. Cancer Epidemiol Biomarkers Prev.

[6] Plummer M, de Martel C, Vignat J, Ferlay J, Bray F, Franceschi S (2016). Global burden of cancers attributable to infections in 2012: a synthetic analysis. Lancet Glob Health.

[7] Zhang J, Zhang Y, Zhang Z (2018). Prevalence of human papillomavirus and its prognostic value in vulvar cancer: A systematic review and meta-analysis. PLoS ONE.

[8] Bleeker MC, Visser PJ, Overbeek LI, van Beurden M, Berkhof J (2016). Lichen sclerosus: incidence and risk of vulvar squamous cell carcinoma. Cancer Epidemiol Biomarkers Prev.

[9] van der Avoort IA, Shirango H, Hoevenaars BM, Grefte JM, de Hullu JA, de Wilde PC (2006). Vulvar squamous cell carcinoma is a multifactorial disease following two separate and independent pathways. Int J Gynecol Pathol.

[10] Schnürch HG, Ackermann S, Alt CD, Barinoff J, Boing C, Dannecker C (2016). Diagnosis, therapy and follow-up care of vulvar cancer and its precursors. Guideline of the DGGG and DKG (S2k-Level, AWMF Registry Number 015/059, November 2015. Geburtshilfe Frauenheilkd..

[11] Nooij LS, Brand FA, Gaarenstroom KN, Creutzberg CL, de Hullu JA, van Poelgeest MI (2016). Risk factors and treatment for recurrent vulvar squamous cell carcinoma. Crit Rev Oncol Hematol.

[12] Clancy AA, Spaans JN, Weberpals JI (2016). The forgotten woman's cancer: vulvar squamous cell carcinoma (VSCC) and a targeted approach to therapy. Ann Oncol.

[13] Helm M, Motorin Y (2017). Detecting RNA modifications in the epitranscriptome: predict and validate. Nat Rev Genet.

[14] Saletore Y, Meyer K, Korlach J, Vilfan ID, Jaffrey S, Mason CE (2012). The birth of the Epitranscriptome: deciphering the function of RNA modifications. Genome Biol.

[15] Vu LP, Pickering BF, Cheng Y, Zaccara S, Nguyen D, Minuesa G (2017). The N(6)-methyladenosine (m(6)A)-forming enzyme METTL3 controls myeloid differentiation of normal hematopoietic and leukemia cells. Nat Med.

[16] Liu J, Eckert MA, Harada BT, Liu SM, Lu Z, Yu K (2018). m(6)A mRNA methylation regulates AKT activity to promote the proliferation and tumorigenicity of endometrial cancer. Nat Cell Biol.

[17] Zhou S, Bai ZL, Xia D, Zhao ZJ, Zhao R, Wang YY (2018). FTO regulates the chemo-radiotherapy resistance of cervical squamous cell carcinoma (CSCC) by targeting beta-catenin through mRNA demethylation. Mol Carcinog.

[18] Strick A, von Hagen F, Gundert L, Klumper N, Tolkach Y, Schmidt D (2020). The N(6) -methyladenosine (m(6) A) erasers alkylation repair homologue 5 (ALKBH5) and fat mass and obesity-associated protein (FTO) are prognostic biomarkers in patients with clear cell renal carcinoma. BJU Int.

[19] Gundert L, Strick A, von Hagen F, Schmidt D, Klümper N, Tolkach Y (2021). Systematic expression analysis of m6A RNA methyltransferases in clear cell renal cell carcinoma. BJUI Compass.

[20] von Hagen F, Gundert L, Strick A, Klumper N, Schmidt D, Kristiansen G (2021). N(6) -Methyladenosine (m(6) A) readers are dysregulated in renal cell carcinoma. Mol Carcinog.

[21] Huang H, Weng H, Chen J (2020). m(6)A Modification in Coding and Non-coding RNAs: roles and therapeutic implications in cancer. Cancer Cell.

[22] Condic M, Ralser DJ, Klumper N, Ellinger J, Qureischi M, Egger EK (2022). Comprehensive Analysis of N6-Methyladenosine (m6A) Writers, Erasers, and Readers in Cervical Cancer. Int J Mol Sci..

[23] Hecking T, Thiesler T, Schiller C, Lunkenheimer JM, Ayub TH, Rohr A (2017). Tumoral PD-L1 expression defines a subgroup of poor-prognosis vulvar carcinomas with non-viral etiology. Oncotarget.

[24] Chesson HW, Dunne EF, Hariri S, Markowitz LE (2014). The estimated lifetime probability of acquiring human papillomavirus in the United States. Sex Transm Dis.

[25] de Martel C, Plummer M, Vignat J, Franceschi S (2017). Worldwide burden of cancer attributable to HPV by site, country and HPV type. Int J Cancer.

[26] Doorbar J (2006). Molecular biology of human papillomavirus infection and cervical cancer. Clin Sci (Lond).

[27] Koh WJ, Greer BE, Abu-Rustum NR, Campos SM, Cho KR, Chon HS (2017). Vulvar Cancer, Version 1.2017, NCCN Clinical Practice Guidelines in Oncology. J Natl Compr Canc Netw..

[28] Dang W, Xie Y, Cao P, Xin S, Wang J, Li S (2019). N(6)-Methyladenosine and Viral Infection. Front Microbiol.

[29] Wu F, Cheng W, Zhao F, Tang M, Diao Y, Xu R (2019). Association of N6-methyladenosine with viruses and related diseases. Virol J.

[30] Wang Q, Guo X, Li L, Gao Z, Su X, Ji M (2020). N(6)-methyladenosine METTL3 promotes cervical cancer tumorigenesis and Warburg effect through YTHDF1/HK2 modification. Cell Death Dis.

[31] Zou D, Dong L, Li C, Yin Z, Rao S, Zhou Q (2019). The m(6)A eraser FTO facilitates proliferation and migration of human cervical cancer cells. Cancer Cell Int.

[32] Geng F, Fan MJ, Li J, Liang SM, Li CY, Li N (2019). Knockdown of METTL14 inhibits the growth and invasion of cervical cancer. Transl Cancer Res.

[33] Li J, Xie H, Ying Y, Chen H, Yan H, He L (2020). YTHDF2 mediates the mRNA degradation of the tumor suppressors to induce AKT phosphorylation in N6-methyladenosine-dependent way in prostate cancer. Mol Cancer.

[34] Ban Y, Tan P, Cai J, Li J, Hu M, Zhou Y (2020). LNCAROD is stabilized by m6A methylation and promotes cancer progression via forming a ternary complex with HSPA1A and YBX1 in head and neck squamous cell carcinoma. Mol Oncol.

[35] Liu L, Wu Y, Li Q, Liang J, He Q, Zhao L (2020). METTL3 Promotes Tumorigenesis and Metastasis through BMI1 m(6)A Methylation in Oral Squamous Cell Carcinoma. Mol Ther.

[36] Hesser CR, Karijolich J, Dominissini D, He C, Glaunsinger BA (2018). N6-methyladenosine modification and the YTHDF2 reader protein play cell type specific roles in lytic viral gene expression during Kaposi's sarcoma-associated herpesvirus infection. PLoS Pathog.

[37] Tsai K, Courtney DG, Cullen BR (2018). Addition of m6A to SV40 late mRNAs enhances viral structural gene expression and replication. PLoS Pathog.

[38] Wyde PR, Ambrose MW, Meyer HL, Zolinski CL, Gilbert BE (1990). Evaluation of the toxicity and antiviral activity of carbocyclic 3-deazaadenosine against respiratory syncytial and parainfluenza type 3 viruses in tissue culture and in cotton rats. Antiviral Res.

[39] Ralser DJ, Condic M, Klümper N, Ellinger J, Staerk C, Egger EK, et al. Comprehensive immunohistochemical analysis of N6-methyladenosine (m6A) writers, erasers, and readers in endometrial cancer. J Cancer Res Clin Oncol. 2022. 10.1007/s00432-022-04083-1. Epub ahead of print. 10.1007/s00432-022-04083-1 PMC10129960 35731272

