# Supplementary material for: N6-methyladenosine RNA modification (m6A) is of prognostic value in HPV-dependent vulvar squamous cell carcinoma
Source: BMC Cancer. 2022 Sep 1;22:943. doi: 10.1186/s12885-022-10010-x (PMC9434921; doi:10.1186/s12885-022-10010-x)
Supplement: Supplementary file 1 — Additional file 1. [file 12885_2022_10010_MOESM1_ESM.pdf]

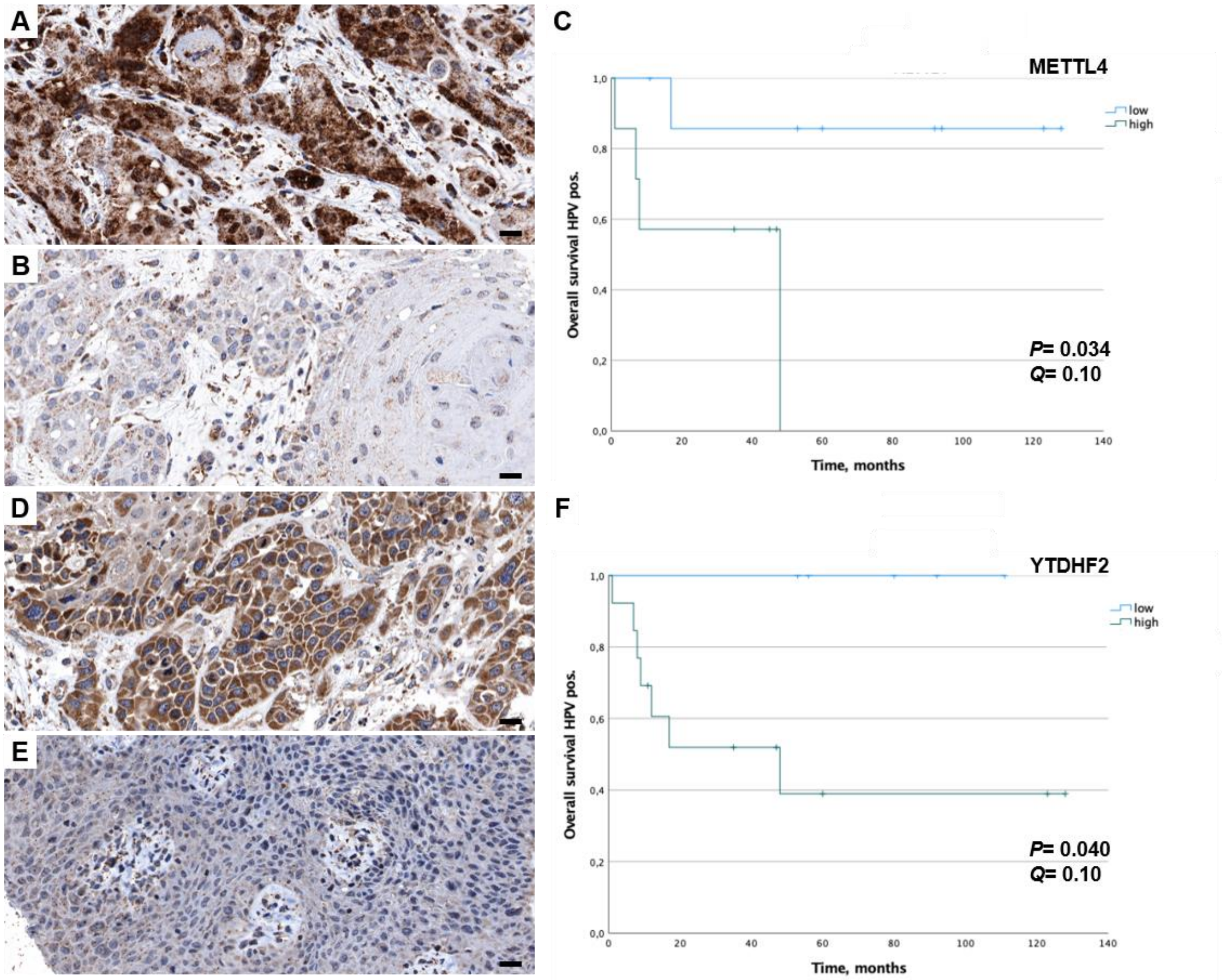

Supplementary Figure 1

Representative histology sections show high (A, D) and low (B, E) expression levels of METTL4 and YTHDF2 visualized by immunohistochemistry; hematoxylin (blue) was used for nuclear staining (bright field image, 400xmagnification). Kaplan-Meier estimates show a significantly shorter 5-year survival ($p<0.05$) in patients with high expression of (C) METTL4, and (F) YTHDF2. There was no prognostic significance after correction for multiple testing ($q<0.1$). Scale bar = 20 um.
