# Supplementary material for: N6-methyladenosine RNA modification (m6A) is of prognostic value in HPV-dependent vulvar squamous cell carcinoma
Source: BMC Cancer. 2022 Sep 1;22:943. doi: 10.1186/s12885-022-10010-x (PMC9434921; doi:10.1186/s12885-022-10010-x)
Supplement: Supplementary file 2 — Additional file 2. [file 12885_2022_10010_MOESM2_ESM.docx]

|  | **Grading** | | | | | | **Lymph node involvement** | | | | | |
| --- | --- | --- | --- | --- | --- | --- | --- | --- | --- | --- | --- | --- |
| **Proteins** | **all** | | **HPV independent** | | **HPV dependent** | | **all** | | **HPV independent** | | **HPV dependent** | |
|  | *N* (G1-G2/G3) | *p*-value | *N* (G1-G2/G3) | *p*-value | *N* (G1-G2/G3) | *p*-value | *N* (N0/>N1) | *p*-value | *N* (N0/>N1) | *p*-value | *N* (N0/>N1) | *p*-value |
| METTL3 | 75 / 26 | 0.087 | 49 / 18 | 0.786 | 13 / 14 | 0.055 | 38 / 30 | 0.780 | 28 / 21 | 0.440 | 2 / 8 | 1.000 |
| METTL4 | 69 / 24 | 0.888 | 48 / 18 | 0.756 | 11 / 3 | 0.710 | 34 / 29 | 0.325 | 29 / 22 | 0.322 | 1 / 6 | 0.857 |
| METTL14 | 70 / 26 | 0.157 | 47 / 18 | 0.348 | 11 / 4 | 0.607 | 34 / 30 | 0.661 | 25 / 21 | 0.699 | 2 / 8 | 0.914 |
| YTHDC1 | 73 / 25 | 0.443 | 49 / 19 | 0.341 | 15 / 3 | 0.102 | 38 / 30 | 0.870 | 28 / 32 | 0.492 | 3 / 8 | 0.376 |
| YTHDF2 | 74 / 27 | 0.458 | 49 / 18 | 0.587 | 13 / 4 | 0.412 | 38 / 31 | 0.773 | 29 / 22 | 0.547 | 2 / 8 | 0.533 |

**Supplementary Table 1.** Correlation analysis of m6A proteins for the entire study cohort, HPV-independent, and HPV-dependent VSCC with grading (G1-G2/G3) and lymph node involvement (N0/>N1) (Mann-Whitney U-test, *p* all >0.05).
